# Individual differences in slow wave sleep architecture relate to variation in white matter microstructure across adulthood

**DOI:** 10.3389/fnagi.2022.745014

**Published:** 2022-08-25

**Authors:** Christel Gudberg, Remi Stevelink, Gwenaëlle Douaud, Katharina Wulff, Alberto Lazari, Melanie K. Fleming, Heidi Johansen-Berg

**Affiliations:** ^1^Wellcome Centre for Integrative Neuroimaging, FMRIB, Nuffield Department of Clinical Neurosciences, University of Oxford, Oxford, United Kingdom; ^2^Nuffield Laboratory of Ophthalmology, Nuffield Department of Clinical Neurosciences, University of Oxford, Oxford, United Kingdom; ^3^Department of Radiation Sciences and Molecular Biology, Umeå University, Umeå, Sweden; ^4^Wallenberg Centre for Molecular Medicine, Umeå University, Umeå, Sweden

**Keywords:** white matter (WM), slow wave (NREM) sleep, electroencephalography (EEG), diffusion imaging, magnetic resonance imaging (MRI)

## Abstract

Sleep plays a key role in supporting brain function and resilience to brain decline. It is well known that sleep changes substantially with aging and that aging is associated with deterioration of brain structure. In this study, we sought to characterize the relationship between slow wave slope (SWslope)—a key marker of sleep architecture and an indirect proxy of sleep quality—and microstructure of white matter pathways in healthy adults with no sleep complaints. Participants were 12 young (24–27 years) and 12 older (50–79 years) adults. Sleep was assessed with nocturnal electroencephalography (EEG) and the Pittsburgh Sleep Quality Index (PSQI). White matter integrity was assessed using tract-based spatial statistics (TBSS) on tensor-based metrics such as Fractional Anisotropy (FA) and Mean Diffusivity (MD). Global PSQI score did not differ between younger (*n* = 11) and older (*n* = 11) adults (*U* = 50, *p* = 0.505), but EEG revealed that younger adults had a steeper SWslope at both frontal electrode sites (F3: *U* = 2, *p* < 0.001, F4: *U* = 4, *p* < 0.001, *n* = 12 younger, 10 older). There were widespread correlations between various diffusion tensor-based metrics of white matter integrity and sleep SWslope, over and above effects of age (*n* = 11 younger, 9 older). This was particularly evident for the corpus callosum, corona radiata, superior longitudinal fasciculus, internal and external capsule. This indicates that reduced sleep slow waves may be associated with widespread white matter deterioration. Future studies should investigate whether interventions targeted at improving sleep architecture also impact on decline in white matter microstructure in older adults.

## Introduction

It is well known that sleep plays a critical role in physical health and cognition (Gudberg and Johansen-Berg, 2015), supporting essential processes of cellular maintenance and restructuring (Vyazovskiy et al., 2007; Tononi and Cirelli, 2014) and helping to preserve and consolidate memory traces over time (Walker et al., 2003; Gudberg et al., 2015). However, sleep has consistently been shown to change considerably across the lifespan, including alterations in broad sleep architecture (Ohayon et al., 2004). In healthy participants, even a single night of sleep deprivation has been shown to lead to significant changes in cognitive function and patterns of electrical activity in the brain during subsequent wakefulness (Hung et al., 2013; Piantoni et al., 2013a). Poor sleep continuity, such as increased sleep fragmentation and wake after sleep onset, has also been associated with a higher rate of cognitive decline, poorer functional outcomes after brain injury, and greater risk of developing Alzheimer’s disease (Lim et al., 2013; Blackwell et al., 2014; Fleming et al., 2020). Evidence from a growing number of studies further suggests that sleep disorders may be strongly linked with more pervasive deterioration in underlying brain structure. For example, sleep disorders, such as insomnia, narcolepsy, and obstructive sleep apnea, have been associated with reduced gray matter volume (Altena et al., 2010; Eun et al., 2010; Koenigs et al., 2010; Joo et al., 2011). Additionally, a recent systematic review (Sanjari Moghaddam et al., 2021) highlighted that white matter microstructure is affected in insomnia, but that the pattern of changes is yet to be understood.

Far fewer studies have considered the relationship between brain structure and sleep microstructure in *healthy* populations. Assessing the association between sleep and variation in white matter microstructure across the lifespan is particularly important as recent evidence suggests a potential role for sleep in the myelination of white matter fibers. For example, gene expression related to the synthesis of myelin has been shown to be preferentially enhanced during sleep and subsequently decreased during wake (Cirelli et al., 2004; Bellesi et al., 2013; de Vivo and Bellesi, 2019), and chronic sleep deprivation has been shown to lead to a reduction in myelin thickness (Bellesi et al., 2018). Evidence is also beginning to emerge from human studies on the relationship between sleep and white matter microstructure (for review see Wassenaar et al., 2019). For example, Grumbach et al. (2020) using data from the Human Connectome Project dataset (healthy young adults), demonstrated that longer subjective sleep duration is associated with higher white matter integrity [Fractional Anisotropy (FA)] in the left superior longitudinal fasciculus. With older adults from the Whitehall II imaging sub-study, Sexton et al. (2017) found that poor subjective sleep quality was associated with globally reduced white matter integrity [i.e., reduced FA, increased axial and radial diffusivity (RD)]. However, this was not replicated by Kocevska et al. (2019a) in their study, which the authors hypothesize may be due to the small effect of subjective sleep complaints on white matter integrity, and objective sleep measures may have a clearer effect. Indeed, a longitudinal study by the same group found that actigraphy measures of sleep continuity (sleep efficiency, wake after sleep onset) relate to global FA and mean diffusivity (MD) measures, whereby poorer sleep quality at one timepoint is associated with worse white matter integrity up to 7 years later (Kocevska et al., 2019b). Together, this supports a hypothesized role for sleep in the maintenance and repair of white matter structural integrity, with a potential to modulate microstructural reorganization over time (Wassenaar et al., 2019).

It is well documented that white matter integrity is decreased with aging (Pfefferbaum et al., 2000; Giorgio et al., 2010; Liu et al., 2016). However, individual variability in the degree of white matter decline observed across age groups (Liu et al., 2016) indicates that factors beyond chronological age may be important mediators of white matter microstructure over time. There are a few studies demonstrating that subjective and objective sleep measures may relate to white matter measures specifically in older adults (Sexton et al., 2017; Altendahl et al., 2020; Aribisala et al., 2020). However, it is unclear whether this relationship is over and above the effects of age. Gaudreault et al. (2018) found that diffusion metrics correlated with frontal sleep spindle amplitude for young adults, but not for an older cohort, suggesting that white matter microstructure may not explain age-related changes in sleep spindle characteristics.

The morphology of sleep slow waves is altered with age (Colrain et al., 2010) and variability in sleep slow wave activity has been shown to be associated with differences in white matter volume in the corpus callosum as well as axial diffusivity (AD) in frontal white matter tracts of young adults (Buchmann et al., 2011; Piantoni et al., 2013b). Measuring the slope of the slow wave is a particularly well-established method for characterizing slow wave morphology, which is thought to provide a sensitive metric of overall sleep homeostasis (Bersagliere and Achermann, 2010; Bouchard et al., 2021) and sleep quality (Tononi and Cirelli, 2014). Steeper slope has been interpreted as a marker of greater synchronized neuronal activity, which has been in turn linked to stronger synaptic connections between neurons (Tononi and Cirelli, 2006; Esser et al., 2007; Huber et al., 2007). Slow wave slope (SWslope) is also known to increase and get steeper during development (Fattinger et al., 2014), and then decrease and become shallower again during aging (Carrier et al., 2011), which has been interpreted as being driven by synaptic strength peaking during adulthood and declining during aging. Taken together with the fact that SWslope has higher temporal resolution than other metrics such as power density (Ujma et al., 2019), we decided to focus our hypotheses and our analyses on the SWslope.

We therefore sought to investigate whether sleep slow wave morphology, specifically the steepness of the rising slope (SWslope), relates to white matter microstructure, and whether this depends on age.

## Materials and methods

### Participants

Twenty-four healthy right-handed adults volunteered to participate. They were characterized as younger (5 males, median age: 25 years, range: 24–27 years) or older (6 males, median age: 61.5 years, range: 50–79 years) and all gave written informed consent to participate in accordance with local ethics committee approvals. Participants filled in a general health questionnaire to check for medication usage, alcohol consumption, caffeine consumption, smoking, and mental health problems. None of the participants had a history of substance abuse, neurological or psychiatric conditions. We excluded anyone with a reported sleep disorder, as well as anyone who used sleep medication or medication known to affect sleep, in order to ensure that we had a sample of healthy adults who did not have sleep problems. This would allow us to probe the extent to which variation in slow wave morphology associates with variation in white matter microstructure in a healthy population, without the confounds of underlying sleep complaints which may have independent effects on white matter microstructure. Two participants dropped out before completing the EEG session and two participants did not complete the sleep questionnaire.

### Assessments

To characterize participants self-reported sleep quality, we used the Pittsburgh Sleep Quality Index (PSQI) (Buysse et al., 1989). This measure assesses participant’s perspective of their sleep quality over the past month, with a total score ranging from 0 to 21 (where a higher score represents poorer sleep quality).

Electroencephalography (EEG) sleep recordings were performed in the participants’ own homes over two consecutive full nights of sleep, with the first night serving as an adaptation night to allow participants to become accustomed to wearing the electrodes during sleep. Participants were asked to abstain from alcohol and caffeine for 12 h before the sleep EEG sessions. EEG, electrooculography (EOG), and submental electromyography (EMG) were recorded by portable biomedical waveform recorders (24 Mbytes, Actiwave, CamNtech). Sixteen electrodes were placed bilaterally on the scalp, above/below the eyes, and around the chin according to a restricted version of the 10–20 system (Klem et al., 1999) and were referenced to the mastoids (right side-A1, left side-A2): F3, F4, C3, C4, P3, P4, O1, O2, EOGl, EOGr. EMG was referenced to the chin. Sample rates were 128 Hz to allow for sufficient overnight recording duration. Sleep stages were subsequently visually scored for 30-s epochs according to the scoring guidelines by Rechtschaffen and Kales (1968). These guidelines were used to allow characterization of individual slow wave stages 3 and 4 non-rapid-eye-movement (NREM) sleep as older adults typically have no stage 4, and reduced representation of stage 3 (Ohayon et al., 2004).

Magnetic resonance imaging (MRI) data were acquired on a 3 tesla Verio scanner (Siemens, Erlangen, Germany) with a 32-channel head coil. Whole-brain diffusion weighted echo-planar images (EPI) were acquired with 60 diffusion directions [repetition time (TR) = 8,900 ms; echo time (TE) = 91.2 ms; diffusion weighting (*b*-value) = 1,500 s/mm^2^; voxel-size = 2 mm^2^; phase-encoding direction: anterior→posterior] plus four volumes without diffusion weighting (*b*-value = 0 s/mm^2^), of which one volume was in the opposite phase-encoding direction (posterior→anterior).

### Analysis

Slow wave detection was performed using a semi-automated MATLAB procedure based on methods outlined previously (Massimini et al., 2004; Piantoni et al., 2013b). Outputs were visually inspected to ensure accuracy and epochs containing artifacts were excluded from analysis. The remaining EEG signal was first low-pass filtered to reduce levels of high-frequency noise in the data. Power spectra for frequencies 0.5–4 Hz were calculated for 30-s epochs and at each electrode position. We focused analyses on values of SWslope in stage 3 NREM sleep, specifically the steepness of the rising slope, derived from frontal electrode sites (right frontal; F4, left frontal; F3) in line with previous findings showing a strong link between frontal white matter and SWslope (Piantoni et al., 2013b), and given that slow waves have been found to preferentially originate in this area (Sekimoto et al., 2000; Murphy et al., 2009).

All imaging analyses were conducted using the FMRIB Software Library (FSL; Jenkinson et al., 2012). Diffusion weighted images were pre-processed using FMRIB’s Diffusion Toolbox (FDT). Topup was used to correct for susceptibility-induced distortions (distortions caused by the magnetic susceptibility inhomogeneities in the subject’s head), using two non-diffusion weighted (*b*-value = 0) images with opposite phase-encoding directions (anterior→posterior and opposite). The susceptibility-induced off-resonance field was estimated using a method similar to that described in Andersson et al. (2003) and the two images were combined into a single corrected one. Next, eddy was used to correct for eddy-current distortion and head movements (Andersson and Sotiropoulos, 2016), and bet was used to exclude any non-brain tissue (Smith, 2002). Finally, dtifit was used to fit a diffusion tensor model at each voxel.

Tract-based spatial statistics (TBSS) was used for voxel-wise comparison of white matter measures of FA, MD, AD, and RD. First, the FA maps of all subjects were non-linearly aligned to a standard FA template (FMRIB58_FA) after which the average was computed. The resulting average FA map was thinned to create a white matter “skeleton,” representing the center of white matter tracts common to all subjects. This white matter skeleton was thresholded at FA > 0.3 to exclude non-white matter and voxels in extremities where there is too much cross-subject variability in alignment, resulting in a white matter skeleton of 100 720 voxels. Next, each subject’s FA data was projected onto the white matter skeleton; for each subject the highest FA value perpendicular to each voxel of the skeleton (i.e., the individual’s local white matter tract center) was projected onto the white matter skeleton. Non-linear warps and skeleton projections estimated based on FA images were then applied to MD, RD, and AD values.

### Statistical analyses

Comparisons of PSQI scores and EEG measures (including SWslope) between the younger and older group were made using Mann Whitney *U*-tests, or independent sample *t*-tests as appropriate (Prism 8, GraphPad Software LLC).

Voxel-wise DTI analyses were performed for participants in whom all assessments had been completed (11 younger, 9 older) using randomize, a tool for permutation-based non-parametric testing using the general linear model (Winkler et al., 2014), as implemented in FSL. We applied a single model to all voxels and all statistical tests were performed with 5,000 unrestricted permutations which is the recommended option for a statistical design such as this (Winkler et al., 2014). Clusters were thresholded for significance using threshold free cluster enhancement (TFCE; Smith and Nichols, 2009), fully corrected for multiple comparisons across space with family-wise error (FWE) correction (corrected *p* ≤ 0.05). All correlations between white matter measures and SWslope were corrected for age group (younger, older) by including group as a regressor in voxel-wise analyses.

## Results

### Sleep and age

**FIGURE 1:**
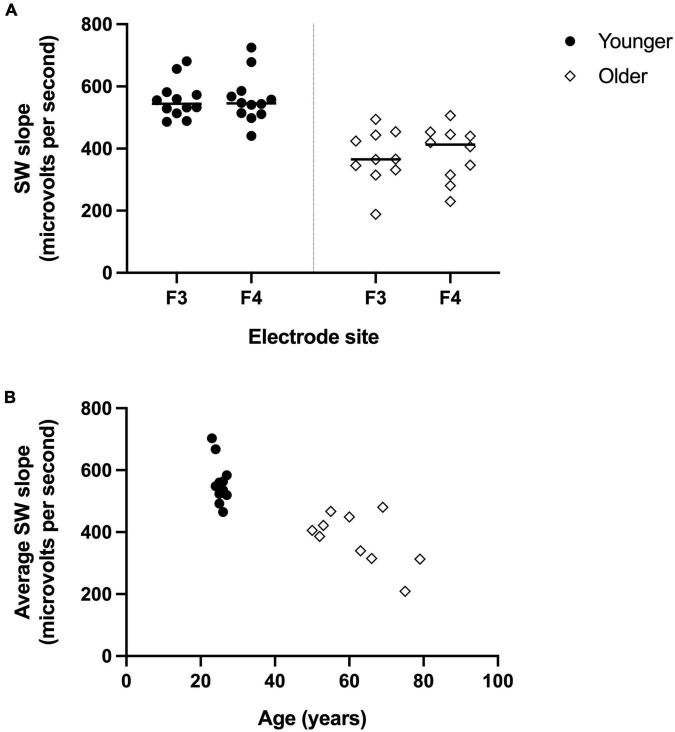
Reduced frontal electrodes slow wave (SW) slope for older adults compared to younger adults. **(A)** SW slope for left frontal electrode (F3) and right frontal electrode (F4) for younger group (filled circles, *n* = 12) and older group (open diamonds, *n* = 10). **(B)** For visualization purposes only, the SW slope (averaged across F3 and F4) is shown for each participant based on their age.

The PSQI and EEG values for the younger and older groups are presented in Table 1. There was no significant difference in self-reported sleep quality between groups (Global PSQI score *p* = 0.505, *n* = 11 per group). For the EEG measures (older *n* = 10, younger *n* = 12), overall sleep time was relatively comparable between age groups (*p* = 0.19), but sleep architecture differed. Older adults spent proportionally more time in stage 2 non-REM (rapid eye movement) sleep (*p* = 0.02) and less time in SWS, with significantly reduced percentage of time spent in stage 3 non-REM sleep (*p* = 0.04) and an absence of stage 4 sleep for the older group. Additionally, SWslope was significantly steeper for the younger group compared with the older at both frontal sites (*p* < 0.001, Figure 1). For visualization purposes only, Figure 1B shows the SWslope averaged across both frontal sites relative to participant’s age.

**TABLE 1:** Self-reported and EEG sleep measures.

	Younger adults	Older adults	Group comparison
**PSQI** *(median, IQR)*			
Global score	2 (1–4)	4 (2–5)	*U* = 50, *p* = 0.51
Quality (1)	0 (0–1)	0 (0–1)	*U* = 60, *p* > 0.99
Latency (2)	0 (0–2)	0 (0–1)	*U* = 53, *p* = 0.61
Duration (3)	1 (0–1)	0 (0–1)	*U* = 47, *p* = 0.39
Efficiency (4)	0 (0–0)	0 (0–1)	*U* = 54, *p* = 0.72
Disturbances (5)	1 (1–1)	1 (1–1)	*U* = 56, *p* > 0.99
Medications (6)	–	–	–
Dysfunction (7)	1 (0–1)	1 (0–1)	*U* = 50, *p* = 0.66
**EEG sleep architecture measures** *(mean, SEM)*			
Total sleep time (hours:minutes)	06:43 (00:13)	07:10 (00:15)	*t* = 1.4, *p* = 0.19
Sleep efficiency (%)	81.5 (2.4)	85.5 (2.4)	*t* = 1.2, *p* = 0.25
Stage 1 (%)	8.3 (1.4)	12.1 (1.7)	*t* = 1.8, *p* = 0.09
Stage 2 (%)	52.7 (1.9)	59.5 (2.1)	*t* = 2.4, *p* = 0.02
Stage 3 (%)	8.6 (1.1)	4.3 (1.6)	*t* = 2.2, *p* = 0.04
Stage 4 (%)	8.3 (1.5)	–	–
REM (%)	22.2 (1.8)	24.1 (1.3)	*t* = 0.8, *p* = 0.42
**SW slope (**μV/s)			
F3 *(median, IQR)*	544.1 (517.7–579.5)	365.6 (327.6–446.6)	*U* = 2, *p* < 0.001
F4 *(median, IQR)*	545.5 (511.8–581.3)	412.8 (307.0–447.7)	*U* = 4, *p* < 0.001

PSQI, Pittsburgh sleep quality index; higher values are indicative of poorer sleep quality: younger n = 11, older n = 11. Electroencephalography (EEG) measures: younger n = 12, older n = 10. SW, slow wave; F3, left frontal electrode; F4, right frontal electrode; IQR, interquartile range; SEM, standard error of the mean. Group comparison is Mann Whitney U-test or t-test as appropriate. Group comparisons are not corrected for multiple comparisons. Note that the values for component 6 of the PSQI (sleep medications) is null as participants were excluded if they were taking medications for sleep. Note that the older group did not show any stage 4 sleep on EEG.

### Sleep and white matter microstructure

**FIGURE 2:**
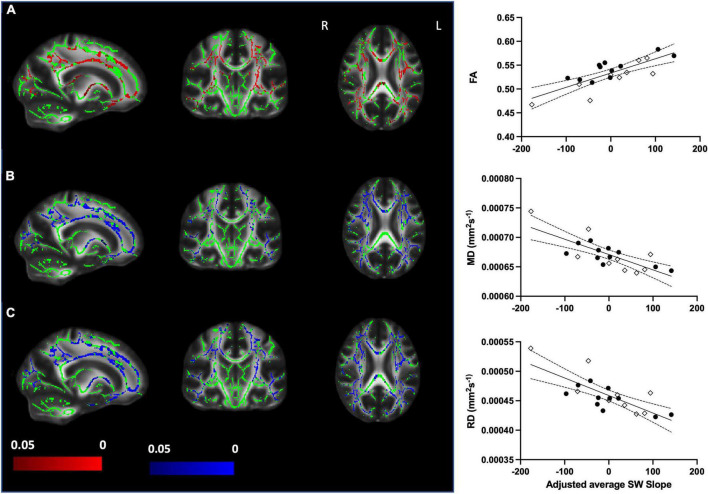
Steeper frontal slow wave slope relates to better white matter integrity when accounting for age group (older *n* = 9, younger *n* = 11). *Left panel*: **(A)** Increased fractional anisotropy (FA) with steeper SWslope. **(B)** Decreased mean diffusivity (MD) with steeper SWslope. **(C)** Decreased radial diffusivity (RD) with steeper SWslope. Images are shown in radiological convention. A significant correlation with SWslope, corrected *p* ≤ 0.05 is shown in red (positive effect) or blue (negative effect). Color bars show *p*-value range. The white matter skeleton is shown in green and overlaid on the FMRIB58 template (FA) brain. There were no significant correlations for AD. *Right panel:* for visualization purposes only, the average individual participants values for FA (top), MD (middle), and RD (bottom) were extracted from the significant voxels (*p* < 0.05) and plotted against average SWslope adjusted for age group. The younger group are shown with filled circles, the older group are shown with open diamonds. The linear regression line and 95% confidence intervals are shown for information purposes only.

We sought to assess whether SWslope related to white matter microstructure across younger (*n* = 11) and older (*n* = 9) adults. Voxelwise analyses revealed that steeper frontal SWslope was associated with higher FA and lower MD/RD, after regressing out age group (Figure 2). However, AD did not show significant correlations with SWslope. For visualization purposes only, we extracted the mean value for each participant over the significant voxels (*p* < 0.05) to plot against frontal SW slope after regressing out age group (Figure 2, right panel). Taken together, these results are indicative of “better” white matter integrity for those with steeper sleep slow waves, across the age groups. If a more conservative threshold was utilized in order to reduce the extent of results (*p* < 0.0125 for FA, *p* < 0.005 for MD/RD) then we can see that the most consistent tracts involved in this association are the corpus callosum and corona radiata (for all measures), the superior longitudinal fasciculus (for MD and RD), the internal capsule (for FA and RD) and the external capsule (for MD). Unthresholded statistical maps are openly available (see data sharing). Similar to the main analyses, for visualization purposes only, we extracted the mean value for each participant over the significant voxels with the conservative thresholds used and plotted these against frontal SWslope after regressing out age group (Supplementary Figure 1).

**FIGURE 3:**
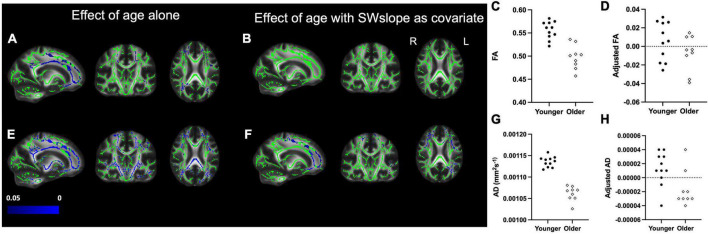
*Left panel:* Decreased fractional anisotropy (FA) for the older (*n* = 9) group compared with the younger (*n* = 11) group **(A)** is eradicated if SWslope is included as a covariate **(B)**. Regions showing decreased axial diffusivity (AD) for the older group **(E)** are reduced in extent when SWslope is included as a covariate **(F)**. Images are shown in radiological convention. A significant negative effect of age group, *p* ≤ 0.05 is shown in blue (color bar shows *p*-value range). The white matter skeleton is shown in green and overlaid on the FMRIB58 template (FA) brain. *Right panel:* for visualization purposes only, the average individual participants values for FA (top) and AD (bottom) were extracted from the significant voxels for the effect of age alone and plotted against age group without **(C,G)** or with adjustment for SW slope **(D,H)**. The younger group are shown with filled circles, the older group are shown with open diamonds.

We also found widespread areas of white matter where older age was associated with lower FA and AD (Figure 3). The differences in FA between age groups did not survive after covarying out SWslope (Figures 3A,B), and differences for AD appear reduced (Figures 3E,F), suggesting that sleep explained a larger proportion of variance in white matter structure, particularly FA, than age group. For visualization purposes only, we extracted the mean value for each participant over the significant voxels for the association between age group and white matter (Figures 3C,G) and the values for the same voxels after regressing out SWslope (Figures 3D,H). There were no significant differences between age groups for either MD or RD.

Due to the variation in age within the older group, for completeness, analyses were repeated with age as a continuous regressor (rather than separating participants into two groups). Findings remained consistent, shown in Supplementary Figures 2, 3.

## Discussion

We found that variation in sleep slow wave morphology was related to individual variation in white matter microstructure in widespread brain areas, over and above the effect of age. This was particularly evident for the corpus callosum, corona radiata, superior longitudinal fasciculus, and the internal/external capsule.

The decline of white matter integrity with aging has been well documented (Pfefferbaum et al., 2000; Giorgio et al., 2010; Liu et al., 2016), but is variable (Liu et al., 2016) and other, potentially modifiable, factors may be important mediators of white matter microstructure over time. Growing evidence on the importance of sleep on brain health and function (Bellesi et al., 2013; Xie et al., 2013; Bernardi et al., 2015; Gudberg and Johansen-Berg, 2015) highlights the potentially critical involvement of sleep in the maintenance of white matter pathways. In the present study, we found significant voxel-wise correlations between frontal SWslope and FA, MD, and RD in widespread white matter tracts, across younger and older age groups. This indicates that steeper sleep slow waves are associated with white matter microstructure metrics reflecting “better” white matter integrity (e.g., higher FA, lower MD, or lower RD), independent of age. We also demonstrated that variation in SWslope explained a larger proportion of the variance in FA across the brain, than did age.

Our findings are supported by a previous study in healthy young, male participants (Piantoni et al., 2013b). Their study showed that SWslope, derived in the same way as in the present study, was significantly correlated with individual differences in white matter microstructure including the corpus callosum and superior longitudinal fasciculus, as seen here, but not for other slow wave parameters. It may be that sleep slow waves significantly correlate with white matter diffusivity due to their reliance on these tracts for slow wave propagation across the brain (Massimini et al., 2004; Murphy et al., 2009). For example, tracts such as the corpus callosum could be particularly important for spreading slow waves across hemispheres (Piantoni et al., 2013b). Here, one hypothesis is that SWslope is indicative of underlying synaptic strength (Esser et al., 2007) and that greater white matter fiber integrity enables greater synchronizity across neurons, reflected as steeper SWslope in the scalp EEG trace. Conversely, it has been hypothesized that sleep slow waves play an important role in the maintenance and repair of brain networks (Tononi and Cirelli, 2006, 2014) and they could therefore also be involved in the support and restructuring of white matter microstructure. In summary, several causal pathways (either alone or in combination) may mediate the observed association between white matter and sleep slow waves across adulthood.

It is also possible that reductions in sleep slow waves are simply a consequence, or comorbid symptom, of other underlying issues such as depression, anxiety, stress, neurodegenerative disease, or obesity, which in turn could be driving the observed differences in white matter microstructure. Similarly, we cannot rule out the possibility that white matter changes are to some extent reflective of age-related cognitive impairments. While we did not specifically assess such factors in this study, and did not perform a specific cognitive assessment, all participants were screened for neuropsychiatric and neurological conditions and were within the normal range for body mass index (BMI). It is nonetheless possible that additional, particularly undiagnosed, health factors could contribute to the observed results. Further research is needed to address the specific contribution of additional lifestyle factors in larger samples.

It is perhaps surprising that the older group did not have poorer self-reported sleep quality. However, there is some suggestion that healthy older adults are less likely to report their sleep quality as poor, even when objectively their sleep is disrupted (Li et al., 2018). Additionally, we deliberately excluded participants with sleep disorders, anyone taking medications specifically for sleep or known to influence sleep quality. Whilst we acknowledge that our sample may therefore not be truly representative of the older adult population, we wanted to probe understanding of the contributions of age and slow wave sleep morphology to variation in white matter integrity without the confound of sleep problems which could have their own influence on brain structure and function. Unfortunately, we did not do a full polysomnography recording and as such we cannot definitively rule out the possibility that any participant could have an undiagnosed sleep condition, such as sleep disordered breathing.

The strongest effects in our results were observed in voxels belonging to a wide range of tracts, including the corpus callosum, corona radiata, superior longitudinal fasciculus, internal and external capsule. These regions, whilst fairly widespread, are consistent with other studies relating white matter microstructure to sleep variables (Piantoni et al., 2013b; Grumbach et al., 2020), including those conducted in older adults (Sexton et al., 2017; Kocevska et al., 2019b) and overlaps with regions previously identified as being altered in insomnia (Li et al., 2016). The widespread reduction in white matter integrity may be driven by effects on multiple systems, from sensorimotor function, which is linked to corpus callosum and internal capsule integrity (Gooijers and Swinnen, 2014; Frizzell et al., 2022), to language function and social cognition, which are linked to the superior longitudinal fasciculus (Madhavan et al., 2014). We used a range of diffusion metrics (FA, MD, RD, AD) to probe different aspects of white matter microstructure. While specific biological interpretation of diffusion metrics can be challenging, particularly in regions of crossing fibers (Figley et al., 2022), our findings suggest a consistent picture of altered white matter microstructure across widely distributed regions.

As such, these findings may simply reflect a distributed effect of sleep on myelination which is independent of specific brain areas. This interpretation is particularly well-aligned with the hypothesized role of sleep in glymphatic clearance of neurotoxic waste and cellular debris (Xie et al., 2013; Fultz et al., 2019), and recent literature that has highlighted whole-brain declines in the glymphatic system with aging (Zhou et al., 2020), as well as changes in gene expressions within precursors of myelinating oligodendrocytes (Shen et al., 2008). Although diffusion metrics such as FA and RD are not specific measures of myelin (Zatorre et al., 2012), it is well established that variations in myelination will modulate both FA and RD (Beaulieu, 2002; Mancini et al., 2020; Lazari and Lipp, 2021). While we cannot rule out the possibility that other changes in white matter microstructure, for example changes in axon diameter, may also contribute to the observed effects, research in rodents provides compelling evidence to suggest that sleep plays a significant role in modulating myelination (Bellesi et al., 2018).

Given the multiple potential interpretations of our results, it will be important for future studies to not only replicate our findings in independent samples and larger populations, but also to further investigate the causal relationships underlying the associations found here to better understand the interaction between sleep and white matter throughout the aging process. Nevertheless, our findings provide further support for interventions targeted at improving sleep quality, which may also impact on age-related decline in white matter microstructure (Castronovo et al., 2014).

## Data availability statement

The datasets presented in this study can be found in online repositories. The names of the repository/repositories and accession number(s) can be found below: Unthresholded statistical maps are available at https://identifiers.org/neurovault.collection:12302 and the commands used to run randomize are provided at https://git.fmrib.ox.ac.uk/alazari/sleep-age-and-microstructure.

## Ethics statement

The studies involving human participants were reviewed and approved by the University of Oxford. The patients/participants provided their written informed consent to participate in this study.

## Author contributions

HJ-B, CG, KW, and GD contributed to the conception and design of the study. CG collected the data and conducted the main data processing and analyses for the study. RS, AL, and MF contributed to further data analysis. CG and MF wrote the draft of the manuscript. AL, HJ-B, RS, KW, and GD contributed to subsequent iterations of the manuscript. All authors contributed to the article and approved the submitted version.
