# Supplementary material for: Individual differences in slow wave sleep architecture relate to variation in white matter microstructure across adulthood
Source: Front Aging Neurosci. 2022 Aug 25;14:745014. doi: 10.3389/fnagi.2022.745014 (PMC9453235; doi:10.3389/fnagi.2022.745014)
Supplement: Supplementary file 1 [file Data_Sheet_1.pdf]

Individual differences in slow wave sleep architecture relate to variation in white matter microstructure across adulthood

Supplementary Figures

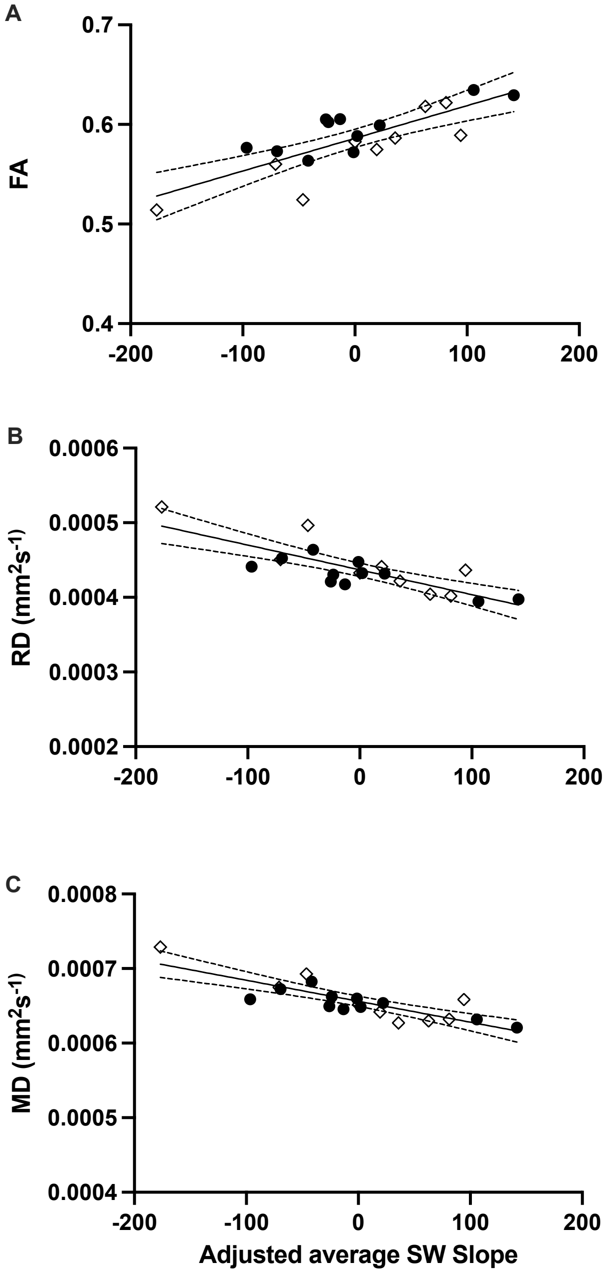

Supplementary Figure S1: For visualization purposes only the average individual participants values for **A:** fractional anisotropy (FA), **B:** radial diffusivity (RD) and **C:** axial diffusivity (AD) were extracted from the significant voxels (FA $p<0.125$, RD/MF $p<0.005$) and plotted against average SWslope adjusted for age group. The younger group (n=11) are shown with filled circles, the older group (n=9) are shown with open diamonds. Linear regression line and 95% confidence intervals are shown for information purposes only.

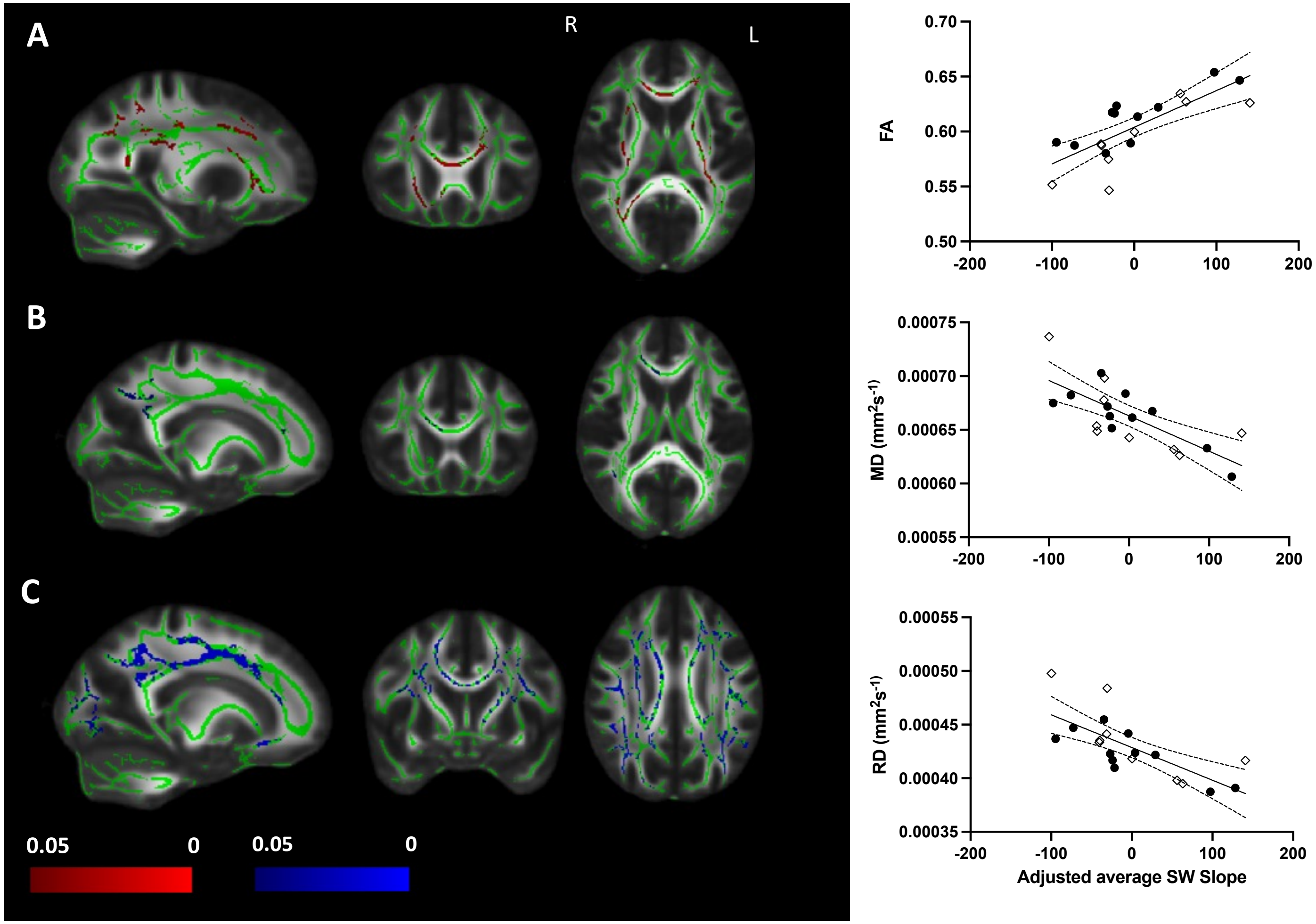

Supplementary Figure S2: Steeper frontal slow wave slope relates to better white matter integrity when accounting for age as a continuous variable (n=20). *Left panel:* **A.** Increased FA with steeper SWslope. **B.** Decreased MD with steeper SWslope. **C.** Decreased RD with steeper SWslope.

Images are shown in radiological convention. A significant correlation with SWslope, $p \leq 0.05$ is shown in red (positive effect) or blue (negative effect). Colour bars show p value range. The white matter skeleton is shown in green and overlaid on the FMRIB58 template (FA) brain. There were no significant correlations for AD. *Right panel:* for visualization purposes only, the average individual participants values for FA (top), MD (middle) and RD (bottom) were extracted from the significant voxels ($p<0.05$) and plotted against average SW slope adjusted for age in years. The younger group are shown with filled circles, the older group are shown with open diamonds. Linear regression line and 95% confidence intervals are shown for information purposes only.

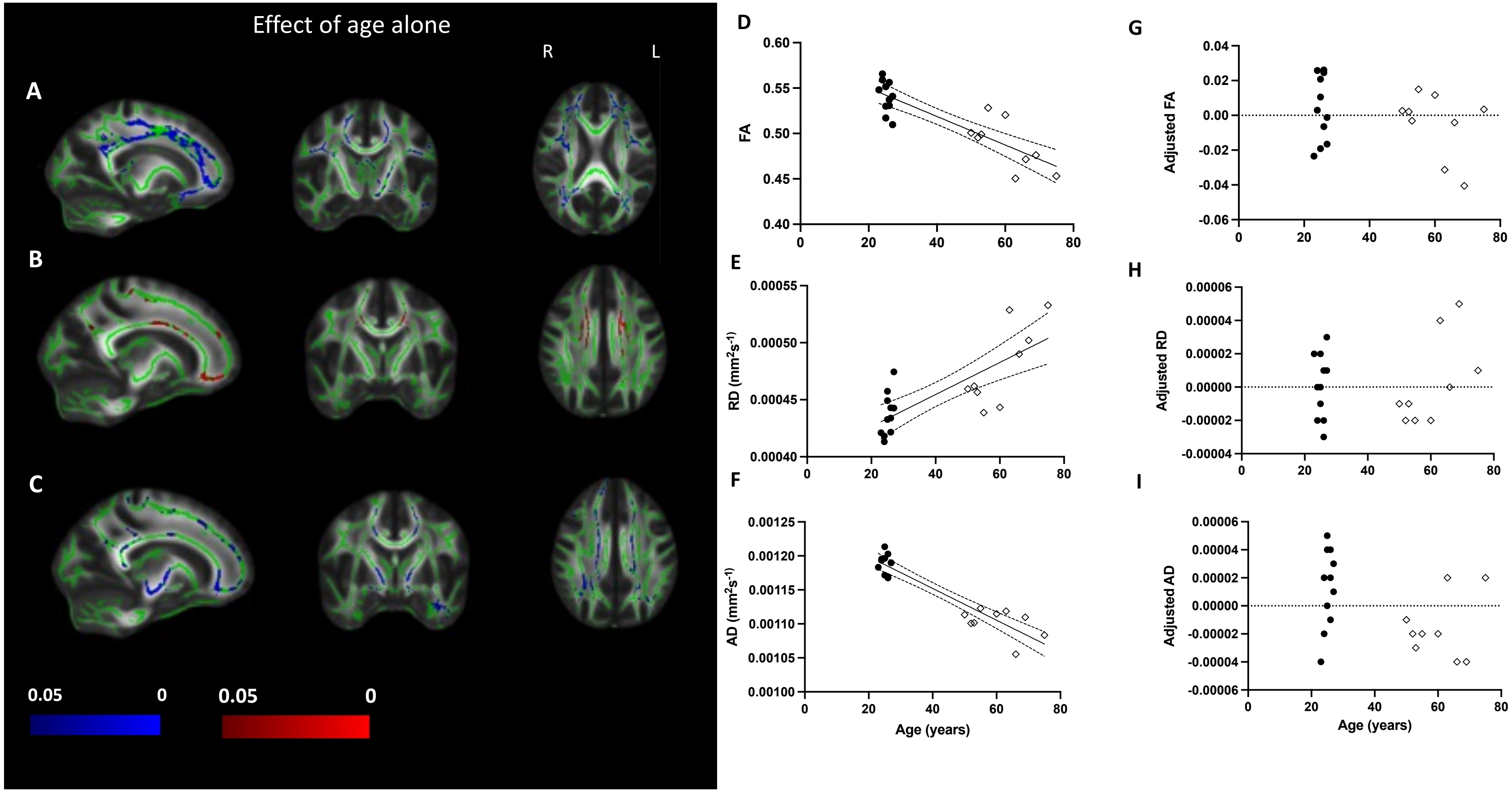

Supplementary Figure S3: *Left panel:* Decreased fractional anisotropy **(A)**, increased radial diffusivity **(B)** and decreased axial diffusivity **(C)** with increased age is eradicated if slow wave slope is included as a covariate (n=20). Images are shown in radiological convention. A significant negative effect of age, p≤0.05 is shown in blue (negative effect) or red (positive effect). The white matter skeleton is shown in green and overlaid on the FMRIB58 template (FA) brain. *Right panel:* for visualization purposes only, the average individual participants values for FA (top), RD (middle) and AD (bottom) were extracted from the significant voxels for the effect of age alone and plotted against age without (D-F) or with adjustment for SW slope (G-I). The younger participants are shown with filled circles, the older participants are shown with open diamonds.
